# Comparative Analysis of Structural Features in SLiMs from Eukaryotes, Bacteria, and Viruses with Importance for Host-Pathogen Interactions

**DOI:** 10.3390/pathogens11050583

**Published:** 2022-05-15

**Authors:** Heidy Elkhaligy, Christian A. Balbin, Jessica Siltberg-Liberles

**Affiliations:** Department of Biological Sciences, Biomolecular Sciences Institute, Florida International University, Miami, FL 33199, USA; helkh002@fiu.edu (H.E.); cbalbin@fiu.edu (C.A.B.)

**Keywords:** short linear motifs, SLiMs, ELM database, intrinsic disordered protein regions, molecular mimicry, protein flexibility

## Abstract

Protein-protein interactions drive functions in eukaryotes that can be described by short linear motifs (SLiMs). Conservation of SLiMs help illuminate functional SLiMs in eukaryotic protein families. However, the simplicity of eukaryotic SLiMs makes them appear by chance due to mutational processes not only in eukaryotes but also in pathogenic bacteria and viruses. Further, functional eukaryotic SLiMs are often found in disordered regions. Although proteomes from pathogenic bacteria and viruses have less disorder than eukaryotic proteomes, their proteins can successfully mimic eukaryotic SLiMs and disrupt host cellular function. Identifying important SLiMs in pathogens is difficult but essential for understanding potential host-pathogen interactions. We performed a comparative analysis of structural features for experimentally verified SLiMs from the Eukaryotic Linear Motif (ELM) database across viruses, bacteria, and eukaryotes. Our results revealed that many viral SLiMs and specific motifs found across viruses and eukaryotes, such as some glycosylation motifs, have less disorder. Analyzing the disorder and coil properties of equivalent SLiMs from pathogens and eukaryotes revealed that some motifs are more structured in pathogens than their eukaryotic counterparts and vice versa. These results support a varying mechanism of interaction between pathogens and their eukaryotic hosts for some of the same motifs.

## 1. Introduction

Protein-protein interactions (PPIs) are pivotal for modulating intracellular processes [1,2]. PPI networks are often regulated through transient interactions, mediated by unstructured protein regions that lack a well-defined structural conformation [3,4,5]. Alteration of PPI networks inside the cell can trigger disease [1,2]. Protein-protein interactions are often mediated by short linear motifs (SLiMs) [6,7,8]. SLiMs are short sequence patterns, with an average length ranging from 3 to 10 sequential residues [5,9,10]. SLiMs can be represented by regular expressions that describe the evolvability of the sequence pattern where amino acid replacements may occur at specific positions, while other positions must be strictly conserved to ensure functionality [5,9,10]. Given the simplicity (the short length and high evolvability) of SLiM sequence patterns, they may occur by chance [11]. When proteins from pathogens display a sequence pattern that matches SLiM motifs in their host, molecular mimicry can result. Through molecular mimicry of SLiMs, pathogen proteins can disrupt native host interactions, often to the benefit of the pathogen. SLiMs are pathogens’ vehicle to hijack and rewire the host interactome [5,8]. 

In 2007, Fuxreiter and coworkers showed that verified SLiMs from the Eukaryotic Linear Motifs (ELM) database were predicted to be mostly intrinsically disordered [10]. The SLiMs were present in more disordered regions with respect to their global surrounding sequence based on disorder prediction. However, the SLiMs themselves, although still disordered, were found to be slightly less disordered than their local adjacent sequence [10]. Intrinsic disorder in proteins refers to a multi-conformational structure with high plasticity and an ability to fold and unfold [12]. The amount of intrinsic disorder can range from small protein regions (intrinsically disordered regions: IDRs) to fully disordered protein (intrinsically disordered proteins: IDPs) [13]. The inherent conformational plasticity of intrinsic disorder allows for conformational changes, which can induce a local structure transition that is essential for a successful protein-protein interaction [12,13]. 

A hurdle in SLiM discovery is the high false-positive rate associated with computational identification approaches. Searching for a SLiM motif using only regular expressions can lead to the discovery of many instances by chance [9,14]. A common approach of reducing false-positive SLiMs is to exclude matches that are not intrinsically disordered [9,15]. However, other studies showed that disorder is not always necessary for the functionality of the motifs [16]. False-positives may also be removed by considering the conservation of the SLiM across homologous proteins [17,18]. While this may work for eukaryotic SLiMs that are more conserved [5], it can prevent the identification of functional SLiMs occurring by chance in regions with high evolutionary rates, such as SLiMs in IDRs [11]. Further, considering only conserved motifs as functional can fail to remove false-positive results occurring in a conserved globular region [19]. Via and coworkers proposed that consideration of surface accessibility and susceptibility to be a loop (to not fold into a secondary structure) could improve the identification of true positive SLiMs, but it may discard buried SLiMs that can be accessible due to allosteric effects [18]. 

We recently analyzed the viral SLiMs from the ELM database, and many of the experimentally verified functional SLiMs were discovered to be devoid of disorder [20]. The lack of disorder in some viral SLiMs is not surprising due to the low disorder content in some viral families [21]. Moreover, eukaryotic proteins include a higher percentage of disordered protein regions than bacteria and viruses [22,23]. Currently, the ELM database contains almost 4000 experimentally verified instances from approximately 300 motifs in proteins from eukaryotes, bacteria, and viruses. SLiMs in the ELM database are divided into six functional categories: cleavage motifs (CLV), degradation motifs (DEG), docking motifs (DOC), ligand-binding motifs (LIG), post-translational modification motifs (MOD), and targeting motifs (TRG) [24]. Due to the growth of the ELM database, reanalysis of SLiMs is essential to highlight differences and similarities between eukaryotes, bacteria, and viruses. Further, a comparison of viral and bacterial SLiMs with their eukaryotic counterparts is warranted to disentangle whether potential differences stem from the taxonomic group or ELM functionality. To this end, we present a comparative analysis of SLiMs from eukaryotes, bacteria, and viruses based on sequence-based predictions of structural characteristics to identify similarities and differences between known SLiMs. 

## 2. Results and Discussion

### 2.1. The Majority of Instances in the ELM Database Bind Ligands and Are from Human 

From the 3934 instances downloaded from the ELM database, 3716 were annotated as true positive SLiMs. Only true positive SLiMs were analyzed, and from hereinafter, SLiMs refer to true positive SLiMs. SLiMs from viruses are often listed as their viral polyprotein product and not as the processed functional protein. Polyproteins can impact the predictions of structural features and thus, we used only viral proteins for which the functional protein product could be determined. Three viral instances were excluded from further analysis since their functional protein could not be resolved.

**Figure 1:**
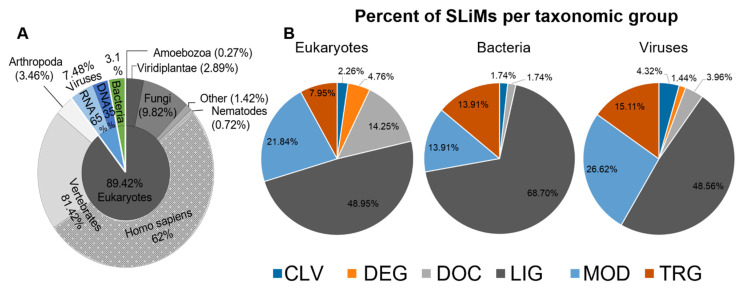
The SLiM dataset composition by taxonomy and functionality. The percentage of SLiMs per taxonomic group and taxonomic subgroup; eukaryotes and its subgroups (grey), viruses and its subgroups (blue), and bacteria (green) based on all SLiMs (**A**). The percentage of SLiMs is colored by functional type in each taxonomic group (**B**). For further information, see Table S1.

In the final dataset of SLiMs used here, the majority of the instances are from eukaryotes (3320 instances), followed by viruses (278 instances) and bacteria (115 instances) (Figure 1A). Most eukaryotic SLiMs are from vertebrates, specifically *Homo sapiens* (2056). The major type of SLiMs represented in all groups is the LIG binding motifs with 1839 SLiMs and the composition of other types varies by taxonomic group (Figure 1B). Although the low number of instances from other taxonomic groups reduces the power of any comparative analysis, to better understand the landscape of SLiMs, we characterize the structural properties associated with the functional type of SLiMs in general and specifically compare SLiMs across taxonomic groups when possible. 

### 2.2. Accessibility and Lack of Secondary Structure Influence SLiM Functionality More than Disorder

**Figure 2:**
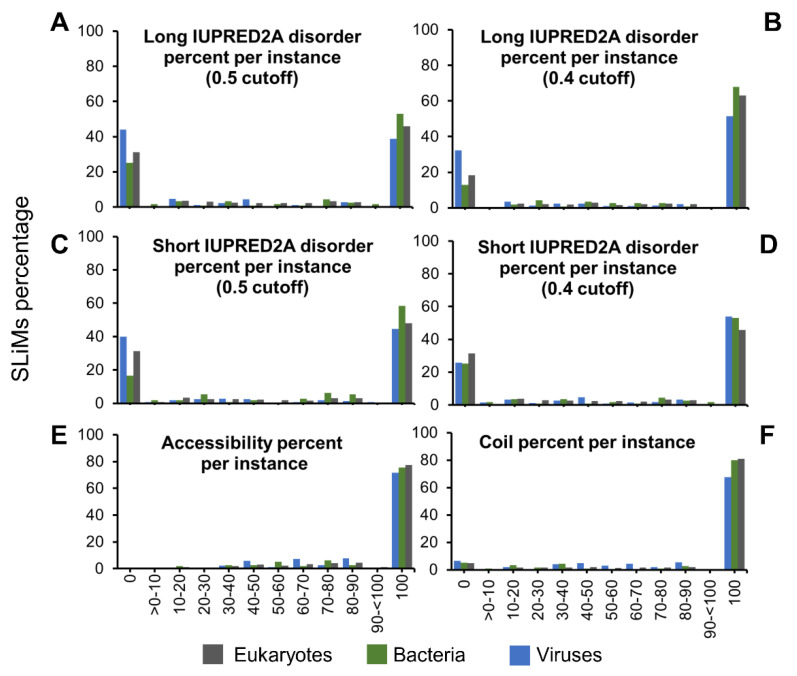
Predicted properties per instance across taxonomic groups. The predicted percentage per instance; IUPRED2A long disorder based on 0.5 cutoff (**A**) and 0.4 cutoff (**B**), IUPRED2A short disorder based on 0.5 cutoff (**C**) and 0.4 cutoff (**D**), NetSurfP 2.0 accessibility based on 0.25 cutoff (**E**), and NetSurfP 2.0 prediction of coil based on three state analysis (**F**). For further information, see Table S1.

To analyze the structural properties of all SLiMs, we predicted intrinsic disorder, surface accessibility, and secondary structure for all residues in proteins with SLiMs in our dataset. The predictions were used to classify each residue within a SLiM as (i) either disordered or ordered based on a cutoff, (ii) either accessible or buried based on a cutoff, and (iii) either in a secondary structure or not in a secondary structure (hereinafter referred to as coil) based on probability from the prediction. The classifications were used to calculate the percentage of disorder, accessibility, and coil, respectively, for each SLiM. Using the default IUPRED2A cutoff (0.5) and the long disorder option to infer disorder, eukaryotes (46%) and bacteria (53%) have a greater proportion of fully disordered instances than viruses (39%) (Figure 2A). Viruses (44%) have a higher percentage of fully ordered instances than eukaryotes (31%) and bacteria (25%). The analysis was repeated with the 0.5 cutoff using the IUPRED2A short disorder prediction option, and the overall trends are the same (Figure 2C). While the IUPRED2A default cutoff for disorder is 0.5, a lower cutoff (0.4) is often used to assign disorder [10]. Using the lower cutoff (0.4), more disordered and less ordered instances were observed with both the long and short IUPRED2A prediction (Figure 2B,D). Few instances have a mixture of ordered and disordered residues for all three groups at both cutoffs (Figure 2A–D). The disorder per SLiM changes for some SLiMs from the long to short IUPRED2A prediction, but the overall correlation is high (Figure S1). In eukaryotes and viruses, the Spearman correlation is high (r_s_ = 0.77 and 0.76, and the *p*-value is 0 and 1.30 × 10^−54^, respectively), while in bacteria, a moderate correlation was observed (r_s_ = 0.52, *p*-value = 1.31 × 10^−9^). For accessibility, we found that eukaryotes (77%) and bacteria (76%) have an increased share of fully accessible instances compared to viruses (71.5%). The remaining percentages vary in percent accessibility per instance in all taxonomic groups (Figure 2E). For coil, eukaryotes and bacteria are similar, with approximately 80% of their instances predicted to be coil (not alpha helix or beta-strand), compared to 68% for viruses. Less than 7% of viral SLiMs and almost 5% of both eukaryotic and bacterial SLiMs were found to have secondary structures (Figure 2D). 

These results reveal that while disorder content varies greatly, accessibility and coil content are prevalent properties across the SLiM distribution. Altogether, these findings suggest a large impact on the functionality of SLiMs for the latter two and an interplay between order and disorder with accessibility and coil. By being fully accessible, SLiMs can interact with other proteins. For partially accessible instances, critical amino acids required for the interaction may be the only exposed residues. Alternatively, the SLiM may be fully or partially concealed until the proper cellular conditions contribute to changing its conformation to become accessible for the interaction to occur. Thus, partially accessible SLiMs could play a pivotal role in regulating the functional cascade triggered by a SLiM. Coil and disorder predictions indicate dynamic, flexible structures for which the conformational ensemble population can vary due to the cellular environment, affecting functional conformations to various degrees. It is plausible that conformational flexibility varies by functionality, such as ELM type. Further, these binary classifications simplify the predictions as a percentage per instance and may not reveal important information about the SLiMs attributes. Exploring the mean IUPRED2A disorder score and mean coil confidence score of SLiMs in each taxonomic category and by ELM type can provide more insights into their structural and functional properties.

### 2.3. SLiMs from Viruses Are Less Disordered 

To explore the mean IUPRED2A disorder score (MIDS) of SLiMs by ELM type, the IUPRED2A disorder prediction scores for all residues within a SLiM were averaged for both long and short disorder predictions, respectively. There is good agreement between long and short disorder prediction overall, but important shifts towards higher disorder from long to short are observed for certain ELM types (Figures S2–S4). A strong positive correlation between long and short disorder predictions that is statistically significant was observed in most of the comparisons by ELM type in each taxonomic group. However, some showed either weak positive correlation, such as DEG in eukaryotes (r_s_ = 0.37, *p*-value = 1.34 × 10^−6^), or moderate correlation such as TRG in eukaryotes (r_s_ = 0.66, *p*-value = 1.26 × 10^−35^) and LIG and TRG in viruses (r_s_ = 0.60 and 0.66, *p*-value = 8.8 × 10^−15^ and 1.74 × 10^−6^, respectively), or no correlation such as MOD in bacteria (r_s_ = 0.03, *p*-value = 0.89). Some motif instances shift from ordered to disordered from the long to the short IUPRED2A prediction, suggesting that not all instances are found in long disordered regions but in short disordered loops. Hence, using only the default long disorder prediction for short viral and bacterial proteins that are known to lack long disordered domains may impact the disorder content of SLiMs and lead to the exclusion of functional motifs in non-eukaryotic pathogens. 

**Figure 3:**
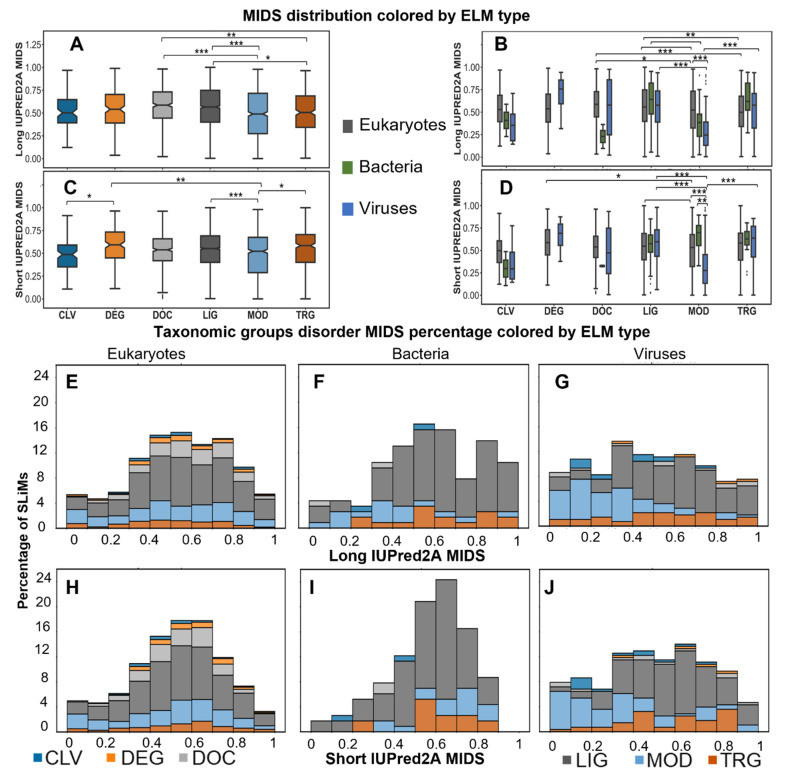
Distribution of MIDS values. Boxplots for the distribution of long IUPRED2A MIDS of all SLiMs per motif type colored as shown by legend (**A**). Boxplots for long IUPRED2A MIDS distribution of all SLiMs in each taxonomic group (bacteria (green), viruses (blue), eukaryotes (grey)) classified based on their ELM type (**B**). Boxplots for the distribution of long IUPRED2A MIDS of all SLiMs per motif type colored as shown by legend (**C**). Boxplots for long IUPRED2A MIDS distribution of all SLiMs in each taxonomic group, colored as in (**B**), classified based on their ELM type (**D**). Hypothesis testing with Mann–Whitney test with simple Bonferroni correction was performed and significant adjusted *p*-values in (**A**,**B**) are shown as brackets between groups (No asterisk for adjusted *p*-values between 0.05 and <0.01, * for adjusted *p*-value ≤ 0.01, ** for ≤1 × 10^−3^, and *** for ≤1 × 10^−4^). The sample size per each tested group and adjusted *p*-values can be found in Table S1. The percentage of SLiMs by long IUPED2A MIDS range in different taxonomic groups colored by ELM type (**E**–**G**). The percentage of SLiMs by short IUPED2A MIDS range in different taxonomic groups colored by ELM type (**H**–**J**), colored as in (**A**). For more information, see Tables S1 and S2.

Hypothesis testing was performed to compare the MIDS distribution for all instances between different ELM types and taxonomic groups. The MIDS values vary greatly between ELM types. For MIDS based on long disorder, MOD and TRG have lower MIDS values than LIG and DOC (Figure 3A). For MIDS based on short disorder, MOD is lower than LIG and TRG, and CLV is lower than DEG (Figure 3C).

Additional MIDS analysis was performed within taxonomic groups by ELM type to investigate differences across taxonomic groups. While most comparisons in both long and short IUPRED2A MIDS are not significant due to the wide dispersion of data within each ELM type, some are significant and may provide insights into the discrepancy of MIDS between ELM types (Figure 3B–D). In viruses, both long and short IUPRED2A MIDS analysis showed that LIG and TRG have higher MIDS values than MOD (adjusted *p*-value = 0 and 7 × 10^−6^, respectively). In bacteria, for the long IUPRED2A MIDS, only LIG is higher than MOD (adjusted *p*-value = 3.86 × 10^−2^). However, there were more observed differences between ELM types in the eukaryotic instances, especially for long IUPRED2A. For DOC, MIDS values are higher than MOD and TRG (adjusted *p*-value = 2.20 × 10^−3^ and 0, respectively) for long IUPRED2A and LIG is higher than MOD and TRG motifs (adjusted *p*-value = 1.85 × 10^−2^ and 4.26 × 10^−4^ respectively) (Figure 3B). For short IUPRED2A MIDS, eukaryotes LIG and DEG were higher than MOD (adjusted *p*-value = 4.41 × 10^−2^, and 3.19 × 10^−3^). The increased MIDS values of the long disorder prediction for DOC and LIG, the main motif types that involve interaction with other proteins inside the cell, support their dynamic role in regulating cellular pathways and machinery and suggests their presence in long disordered regions rather than short disordered loops. Comparing MIDS for the same ELM type across taxonomic groups revealed that the only difference between taxonomic groups was for MOD motifs in viruses, which had lower MIDS than eukaryotes (adjusted *p*-value = 0) for both long and short disorder, and bacteria (adjusted *p*-value = 8.52 × 10^−4^) for short disorder.

To further explore the distribution of MIDS by taxonomic group, the percentage of SLiMs per ELM type across different MIDS ranges were plotted (Figure 3E–J). Based on long disorder analysis of the MIDS values and a 0.4 cutoff, approximately 73% and 77% of the instances in eukaryotes and bacteria, respectively, are disordered, while approximately 59% of the viral instances are disordered. Proportionally, the amount of each ELM type appears similar across MIDS bins for all taxonomic groups, except MOD in bacteria and viruses, which have lower MIDS values (Figure 3E,F). For the short IUPRED2A disorder analysis of MIDS, the amount of disordered eukaryotic SLiMs is similar to MIDS from long disorder, while viruses and bacteria show an increased amount of disordered SLiM for MIDS from short disorder (64% and almost 83%, respectively). Most MOD instances from viruses and bacteria have lower MIDS values than eukaryotes for long IUPRED2A prediction (Figure 3E,F). For short IUPRED2A prediction, MOD in bacteria is more disordered, and viruses show a subtle shift towards disorder for some instances (Figure 3I,J). Notably, the number of instances in the highest MIDS category based on long disorder is reduced for bacteria and viruses for short disorder (Figure 3I,J). An analysis of MODs from the same motifs from different taxonomic groups is required to generalize or discard this trend. 

### 2.4. Most SLiMs Lack Secondary Structure

To explore the mean coil confidence score (MCCS) of SLiMs by ELM type, the NetSurfP coil confidence scores for all residues within a SLiM were averaged. Hypothesis testing was performed to compare the MCCS distribution for all instances between different ELM types and taxonomic groups. All data have a negatively skewed distribution with the highest percentages of SLiMs in the upper bin range of 0.9 to 1 MCCS values. Analyzing the SLiMs MCCS by ELM type revealed that DOC has higher MCCS values than CLV and MOD (adjusted *p*-value = 1.25 × 10^−2^ and 7.1 × 10^−3^, respectively). DEG also shows an increase in MCCS values compared to CLV and MOD types (adjusted *p*-value = 3.19 × 10^−3^, and 6.24 × 10^−3^, respectively) (Figure 3A). Intrinsic disorder properties of proteins have previously been linked to proteins being unstructured or having enough plasticity to undergo structural transitions [25,26]. While most SLiMs are predicted to have high coil confidence, instances from some ELM types show great variation in MCCS values. This may indicate a presence of structural transitions and spatiotemporal control of the structure to perform the function of the SLiMs, but it may also indicate that some SLiMs are not conformationally flexible but lack disorder and have secondary structure. 

**Figure 4:**
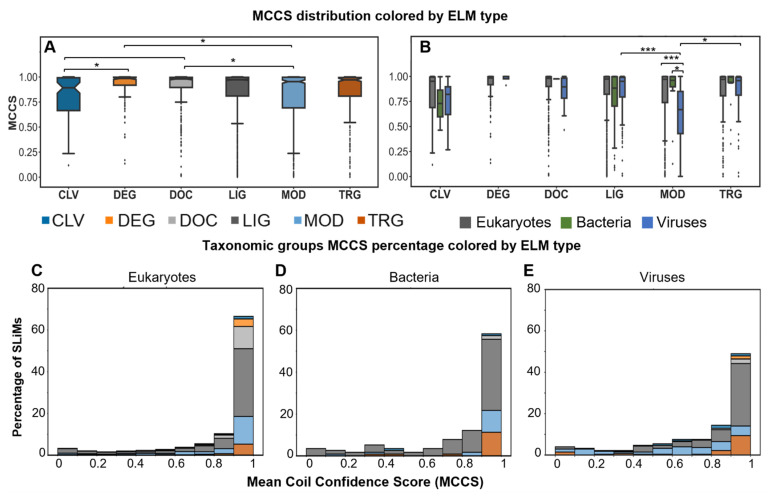
**Distribution of MCCS values.** Boxplots for the distribution of MCCS of all SLiMs per motif type colored as shown by legend (**A**). Boxplots for MCCS distribution of all SLiMs in each taxonomic group (bacteria in green, viruses in blue, and eukaryotes in grey) classified based on their ELM type (**B**). Hypothesis testing with Mann–Whitney test with simple Bonferroni correction was performed and significant adjusted *p*-values in (**A**,**B**) are shown as brackets between groups (No asterisk for adjusted *p*-values between 0.05 to <0.01, * for adjusted *p*-value ≤ 0.01, and *** for ≤1 × 10^−4^). The sample size per each tested group and adjusted *p*-values can be found in Table S1. The percentage of SLiMs by MCCS range in different taxonomic groups colored by ELM type (**C**–**E**) colored as in (**A**). For more information, see Tables S1 and S2.

Instances analysis of MCCS across taxonomic groups highlighted great variability in CLV and LIG for all groups. In MOD, only viruses showed great variability. In viruses, MCCS values for MOD motifs were lower than LIG and TRG (adjusted *p*-value = 0 and 2.27 × 10^−3^, respectively). Comparing scores for MOD between taxonomic groups found viruses lower than eukaryotes and bacteria (adjusted *p*-value = 0 and 5.04 × 10^−3^, respectively) (Figure 4B). 

To further explore the distribution of MCCS by taxonomic group, the percentage of SLiMs per ELM type across different MCCS ranges were plotted (Figure 4C–E). The analysis of SLiMs percent distribution of MCCS in all taxonomic groups revealed that most SLiMs have high coil confidence (Figure 4C–E). Approximately 80% in eukaryotes, 60% in bacteria, and 50% in viruses have MCCS > 0.9. The LIG motifs are the predominant motifs with MCCS > 0.9. MOD sites have higher distribution over all MCCS ranges of viral instances than other taxonomic groups. Altogether, the lower values and the great variability in MCCS of viral MOD sites support the MIDS results, suggesting that some modification sites, especially in viruses, are ordered (not disordered or coil).

### 2.5. Disordered or Flexible? 

The above-mentioned results led us to pose three questions. First, we asked whether SLiMs possess intrinsic disorder and coil properties that differ from of the overall protein context. Second, we asked if the same SLiMs from different taxonomic groups are different from each other. Third, for the shared motif instances, is there any variation in their structure-based sequence properties that might affect the functionality between different groups?

#### 2.5.1. SLiMs Are Found in Flexible Regions

**Figure 5:**
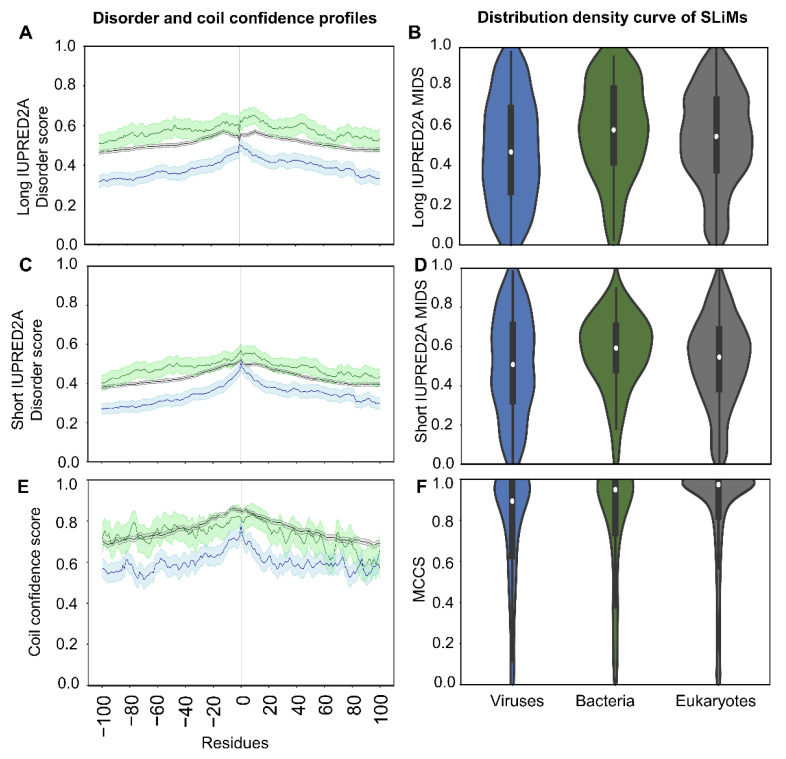
Disorder and coil confidence profiles of proteins containing SLiMs and the density curve of MIDS and MCCS of SLiMs per taxonomic group. The flanking regions of 100 residues around SLiMs using long IUPRED2A disorder score per taxonomic group and the 95% confidence interval of the mean (**A**). SLiMs long IUPRED2A MIDS density distribution plot of the SLiMs per taxonomic group (**B**). The flanking regions of 100 residues around SLiMs using short IUPRED2A disorder score per taxonomic group and the 95% confidence interval of the mean (**C**). SLiMs short IUPRED2A MIDS density distribution plot of the SLiMs per taxonomic group (**D**). The flanking regions of 100 residues around SLiMs coil confidence score per taxonomic group and the 95% confidence interval of the mean (**E**). SLiMs MCCS density distribution plot of the SLiMs per taxonomic group (**F**). For further information, see Table S3.

To answer the first question of whether SLiMs differ in intrinsic disorder content and in secondary structure compared to the overall protein context, we extracted the long and short disorder and coil confidence scores for the flanking regions of each instance. We examined the 100 residues before and after the SLiM instance in each taxonomic group. The mean of all positions and the 95% confidence interval were computed and plotted with the center (zero) representing the mean long or short MIDS or MCCS values per instance for all instances, based on long mean MIDS (mMIDS), short mMIDS, and mean MCCS (mMCCS), respectively (Figure 5A,C). 

For disorder, instances from the three taxonomic groups show the same overall trend with increasing MIDS values towards mMIDS in both long and short disorder IUPRED2A predictions, but with less disordered flanking regions in viruses than in eukaryotes and bacteria (Figure 5A). Bacterial instances are more disordered than eukaryotic instances; however, due to the wide MIDS confidence interval range for the bacterial instances, this might not hold true if more data are explored. Viral instances have the lowest mMIDS values between tested groups using both long and short disorder predictions, supporting previous findings that viruses can be more ordered than other taxonomic groups [22,23]. In addition, using the long IUPRED2A disorder, the bacterial and eukaryotic SLiMs are located in a less disordered region than their immediate surrounding region (Figure 5A), in agreement with previous work [10]. The effect is more prominent in eukaryotes, where a crater-like dip in disorder surrounds the mMIDS and may change for bacteria if more data was available. When SLiMs are located in a less disordered protein region than the surrounding region, the highly disordered surrounding regions can regulate or enhance the binding of the SLiM in protein complexes in accordance with protein fuzziness [27,28]. We observe no such pattern of a dip in mMIDS for SLiMs compared to the flanking region in viruses. Moreover, no dip was observed when using the short IUPRED2A prediction, where mMIDS for all taxonomic groups are in an overall more disordered region than the flanking region (Figure 5A–C). It should be noted that due to limited data availability for both virus and bacterial instances in the ELM database, the confidence intervals in viral and bacterial results have higher uncertainty than in eukaryotes. 

The density curve for long disorder MIDS per SLiM per taxonomic group demonstrates a higher density above the cutoff 0.4 for eukaryotes and bacteria while viruses reveal almost equal density for the entire range. For both eukaryotes and bacteria, the density becomes more centered around the median for the short disorder MIDS per SLiM, with the highest density of SLiMs found at approximately 0.6 IUPRED2A short disorder value. For viruses, two subtle peaks of high density of instances appeared at approximately 0.4 and 0.6 for the short disorder MIDS per SLiM (Figure 5B–D). 

For all taxonomic groups, MCCS of flanking regions increases towards mMCCS of SLiMs (Figure 5E). SLiMs from eukaryotes and bacteria have relatively similar mMCCS values, while SLiMs from viruses have lower mMCCS values. The flanking regions in viruses have lower MCCS than eukaryotes and bacteria (Figure 5C). The density curve for MCCS per SLiMs per taxonomic group shows that viruses closely resemble bacteria above 0.8. The corresponding density plot for eukaryotes has a high density near the maximum MCCS (Figure 5D).

#### 2.5.2. A Comparison of Viral and Bacterial Motifs with Their Corresponding Eukaryotic Motifs 

**Figure 6:**
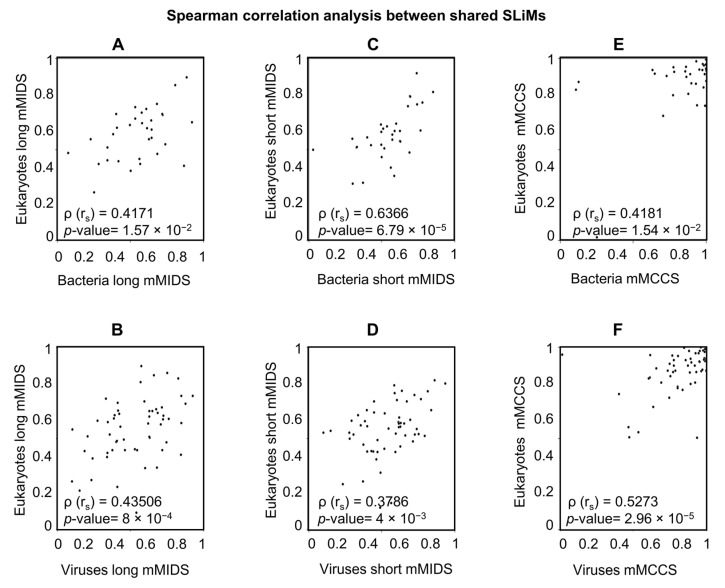
Scatter plot for the MIDS and MCCS means of the shared SLiMs between different groups. Long disorder MIDS means scatter plot and Spearman correlation with the *p*-value for shared SLiMs between eukaryotes vs. bacteria (**A**) and eukaryotes vs. viruses (**B**). Short disorder MIDS means scatter plot and Spearman correlation with the *p*-value for shared SLiMs between eukaryotes vs. bacteria (**C**) and eukaryotes vs. viruses (**D**). MCCS means scatter plot and Spearman correlation with the *p*-value for shared SLiMs between eukaryotes vs. bacteria (**E**) and eukaryotes vs. viruses (**F**). For detailed information about the number of instances, long/short mMIDS and mMCCS of all instances per motif, long/short MIDS and MCCS per instance, and the individual amino acid scores of disorder and coil confidence per instance, see Table S4.

To answer the second question, if the same SLiMs from different taxonomic groups are different from each other, a correlation analysis of the disorder scores or coil confidence for SLiMs from one taxonomic group versus the equivalent SLiMs in another taxonomic group was performed. Motifs shared between taxonomic groups were extracted to compare corresponding sequence-based structural properties. Viruses and bacteria share only 16 motifs, and no further analysis was performed due to the low number of instances. Viruses and eukaryotes share 56 motifs. Bacteria and eukaryotes share 33 motifs (For detailed information see, Table S4). Due to differences in the number of instances between groups for each motif, the mMIDS (long/short) and mMCCS for all instances of a motif were calculated and used to infer the correlation between shared motifs with Spearman correlation analysis. The correlation analysis for the shared motifs revealed a moderate or strong positive correlation with a significant *p*-value for all tested pairs (Figure 6). 

The mMIDS for most shared motifs are in good agreement between the compared groups (eukaryotes and viruses or bacteria) (Table S4). For the shared motifs that were not in good agreement, the individual MIDS and MCCS of each instance and the disorder/coil confidence score per residue were inspected. Some motifs showed a considerable variation in the MIDS and MCCS values of instances and the individual amino acid disorder and coil confidence scores. The variability in disorder scores across a motif has previously been explained by the functionality of each residue within the motif [10]. Eukaryotic motifs’ wide range of MIDS and MCCS values may be influenced by numerous factors, such as the species and protein where the SLiM is found, its potential interacting protein partner, the proposed function of SLiM (to regulate the function or to activate or inhibit the function of the interacting protein permanently), the dynamics of the interaction or the interacting context in which the SLiM-protein interaction occurs (i.e., the energy of the interaction of the motif and the surrounding sequence with the interacting protein). The variability in MIDS and MCCS scores and the existence of different factors affecting SLiM interaction supports a dynamic nature of SLiMs interactions with their target protein in real-time and on evolutionary time scales as well as mutational processes. 

#### 2.5.3. To Fold or Not to Fold: A Tale of Two Motifs 

To investigate our third question about the shared motif instances, is there any variation in their structure-based sequence properties that might affect the functionality between different groups? Two shared motifs between viruses and eukaryotes, MOD_N-GLC_1 and LIG_Rb_LxCxE_1, were selected for further analysis. The first motif, MOD_N-GLC-1, makes up almost 80% of all viral MOD motifs, and it is the most abundant ELM type in viruses below the 0.4 cutoff in both long and short IUPRED2A results. Overall, this motif is devoid of disorder, with long mMIDS below 0.4 for both eukaryotes and viruses, 0.27 and 0.23, respectively, and nearly similar values for the short mMIDS values as well (Table S4). The second motif, LIG_Rb_LxCxE_1, makes up approximately 10% of all viral LIG motifs and is the most abundant LIG ELM type in viruses below the 0.4 cutoff in both disorder prediction types. The long IUPRED2A mMIDS for viruses (0.37) suggests more ordered instances, while the long IUPRED2A mMIDS for eukaryotes (0.43) suggests more disordered instances. However, the short IUPRED2A mMIDS data for viruses showed a slightly higher value of 0.42, and eukaryotes had almost equivalent value to the long IUPRED2A mMIDS value (Table S4), although this differentiation is not meaningful as all are in a similar range. Both motifs had a considerable number of instances in viruses and eukaryotes that enabled further sequence and structure investigation to discover potential differences or similarities between these two groups.

##### Are MOD_N-GLC_1 Instances Indeed Predominantly Ordered in Viruses or Is This Perhaps Due to Insufficient Data?

The MOD_N-GLC-1 motif has the regular expression pattern. (N)[^P][ST].. in the ELM database of where (dot) means any amino acid is accepted at this position, (N) means only asparagine is accepted, [^P] means any amino acid except proline is accepted, and [ST] means that only serine or threonine are accepted at this position [24]. Oligosaccharyl transferase recognizes the pattern and results in N-linked glycosylation on the asparagine residue (N) at the beginning of the motif in unfolded proteins [24,29,30]. Glycosylation is a post-translational modification that usually aids in protein folding. The glycosylated protein region may acquire a specific fold or be a part of the structured domain, or remain a coil [31]. Viral proteins are glycosylated by the glycosylation enzymes of their host [30]. Glycosylation has a wide range of effects on viruses, such as altering viral protein folding and function, inducing interactions with glycan-binding proteins, assisting immune cell evasion, pathogenicity, cellular tropism, and blocking access to other functional regions (reviewed in [30]). 

**Figure 7:**
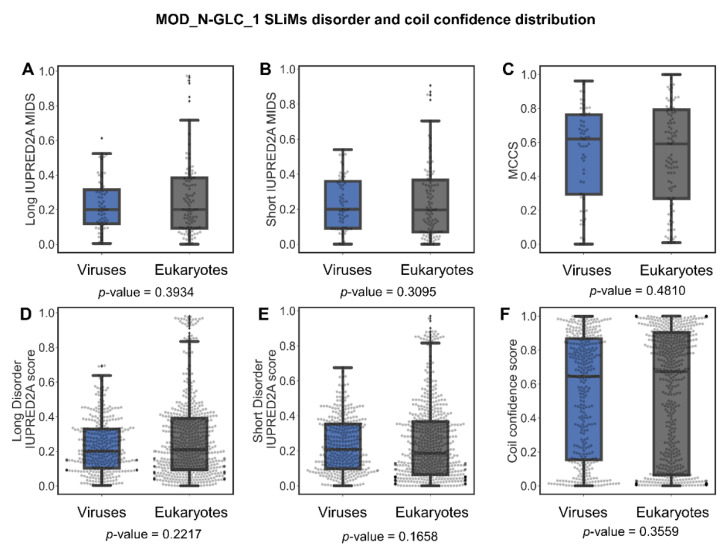
Disorder score and coil confidence distributions in viruses and eukaryotes for the MOD_N-GLC_1 motif. Boxplots and swarm plot distribution for SLiMs long IUPRED2A MIDS (**A**), short IUPRED2A MIDS (**B**), MCCS (**C**), the individual long IUPRED2A disorder scores per residue for SLiMs (**D**), the individual short IUPRED2A disorder scores per residue for SLiMs (**E**), and the individual coil confidence scores per residue for SLiMs (**F**).

There were 156 MOD_N-GLC_1 instances in the ELM database, 59 from viruses and 97 from eukaryotes. Further analysis was performed for the distributions of long and short MIDS, disorder score values, MCCS values, and coil confidence values per amino acid residue. The comparisons revealed no difference between eukaryotes and viruses (Figure 7). For both groups, the variation in coil confidence reaches from 0 to 1 with an accumulation at both ends. The range of coil confidence and the low disorder score of this motif may be due to the glycosylation effects. Glycosylation occurs in the endoplasmic reticulum co- or post-translationally and may induce folding of these specific sites in the protein [32,33,34,35]. Although the MOD_GLC-1_N motif instances shared between eukaryotes and viruses are often ordered, some are found to be disordered. One of the MOD_GLC-1_N motifs that is predicted to be disordered is the West Nile virus motif, which also has an annotated 3D structure in the ELM database.

**Figure 8:**
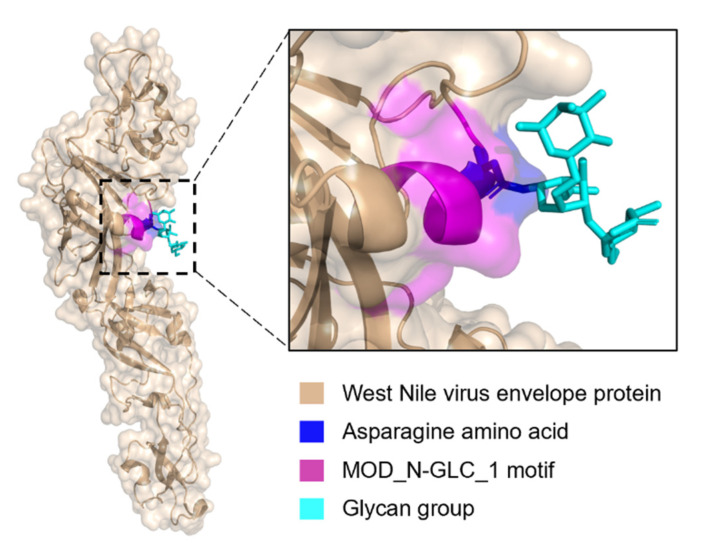
The glycosylated MOD_N-GLC_1 site in West Nile virus envelope protein. West Nile Virus envelope protein (beige) (PDB ID: 2HG0) rendered as a transparent surface. A closer view of the local helical structure of the MOD_N-GLC_1 motif (magenta). The glycosylated asparagine residue (blue) and glycan group (cyan) are shown as sticks.

The annotated structure from the ELM database is for the West Nile Virus (WNV) envelope protein with the MOD_N-GLC_1 motif in region 443 to 448 in PDB ID: 2HG0 [36]. Based on reduced DSSP [37] assignments of this structure, the secondary structure for the motif region is a mixture of coil and helix (CCHHHH) (Figure 8). This is different from the prediction for this instance, which is 100% coil with an MCCS value of 0.81, a MIDS score of 0.52 for long IUPRED2A prediction, and a MIDS score of 0.45 for short IUPRED2A prediction. In another structure of the same protein but without the glycosylation (PDB ID: 3I50), this site is not resolved in the structure [38]. Unresolved, missing residues in structures from X-ray crystallography indicate disordered regions [39,40,41]. The MOD_N-GLC_1 motif in the envelope protein from WNV that is flexible and accessible in the nascent initially unfolded state facilitates glycosylation by the host’s Oligosaccharyl transferase. Upon glycosylation and the protein folding, the motif transitions to a more rigid (less flexible) conformation. This example illustrates how the structural states are context-dependent. 

**Figure 9:**
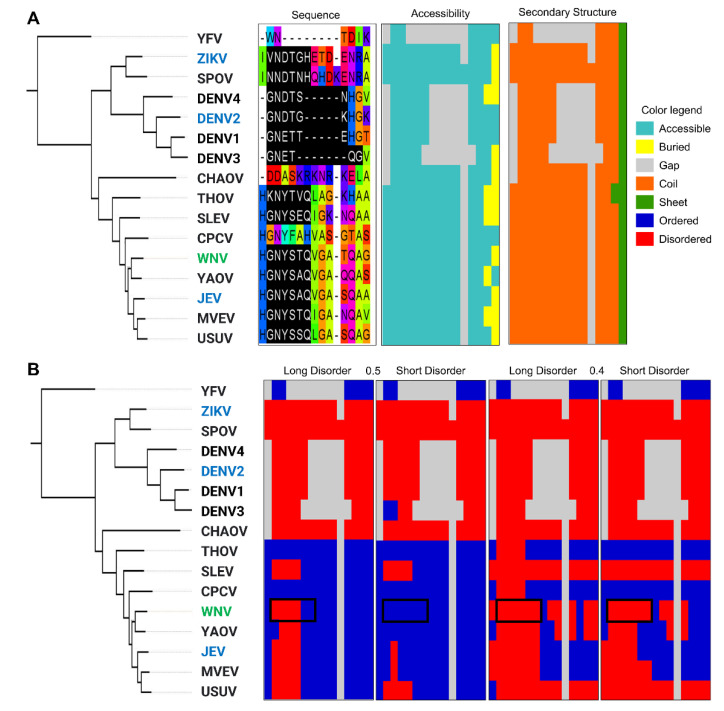
Phylogenetic tree of West Nile Virus (WNV) envelope protein illustrating the evolution of structural properties of a MOD_N-GLC_1 motif. The tree, rooted by the outgroup Yellow Fever virus (YFV)), shows WNV in green and Zika virus (ZIKV), Dengue virus 2 (DENV2), and Japanese Encephalitis Virus (JEV) that have been shown to be glycosylated in this position but that are not in the ELM database in blue. The tree is shown next to an excerpt from the multiple sequence alignment with the MOD_N-GLC_1 motif pattern highlighted in black, followed by the same alignment excerpt colored by the accessibility and secondary structure of the residues (**A**) and by disorder using both 0.5 and 0.4 cutoff values for long IUPRED2A and short IUPRED2A disorder, with the location of the WNV MOD_N-GLC_1 motif shown by the black box (**B**). For further details, see Figure S5.

A small phylogeny was constructed for the envelope from WNV and its homologs. Sequence-based structure properties predictions were performed and mapped to the multiple sequence alignment. The N-glycosylation site from the MOD_N-GLC_1 motif is mostly conserved, but the whole motif is missing from two close relatives of WNV and from the Yellow Fever virus (YFV) outgroup (Figure 9). For the viruses that harbor the MOD_N-GLC_1 motif in this region, all display similar patterns of accessibility and coil, but the amount of disorder in the region varies. Although WNV envelope protein is the only annotated motif of this type in the ELM database, other viruses such as Zika, Dengue, and Japanese Encephalitis viruses were found to be glycosylated at the same alignment site, and its glycosylation has been found to increase infectivity [42,43,44,45,46,47]. It is plausible that variation in flexibility of this region can impact glycosylation between viruses and, consequently, their infectivity. 

##### LIG_Rb_LxCxE_1 Is Less Disordered in Viruses

The LIG_Rb_LxCxE_1 motif is an amino acid sequence with a pattern shortly represented as LxCxE [48]. This motif is recognized by the tumor suppressors retinoblastoma protein (Rb), p107, and p130 involved in impeding G to S phase cell cycle progression [49]. Rb inhibits gene transcription through interactions with LIG_Rb_LxCxE_1 on the transcription factor E2F. Phosphorylation of the Rb protein initiates the release of E2F, and subsequently E2F-DNA binding activates cell cycle progression [48,49,50,51]. The LIG_Rb_LxCxE_1 motif has been found in viruses, especially DNA viruses [48,49,51,52,53]. Viral proteins displaying the LIG_Rb_LxCxE_1 motif bind to the Rb protein and leave the E2F transcription factor able to stimulate the cell cycle progression. Once cells replicate, the viruses take advantage of the replication enzymes to replicate their genome [54,55,56]. 

**Figure 10:**
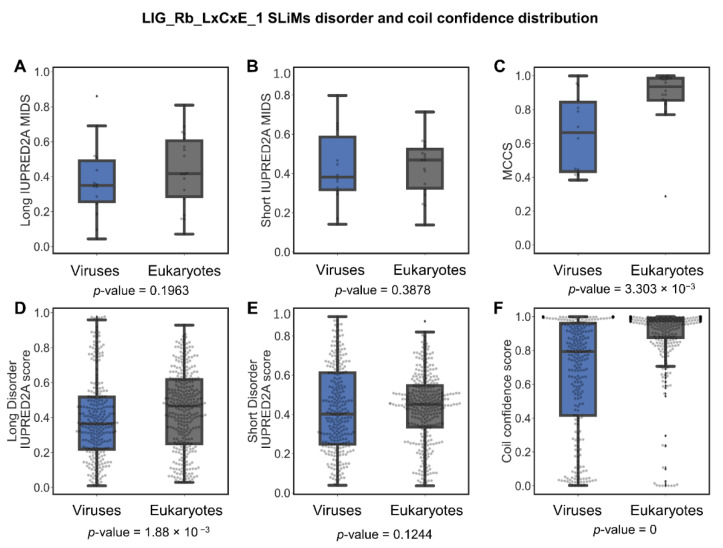
Disorder score and coil confidence distributions in viruses and eukaryotes for the LIG_Rb_LxCxE_1 motif. Boxplots and swarm plot distribution for SLiMs long IUPRED2A MIDS (**A**), short IUPRED2A MIDS (**B**), MCCS (**C**), individual long IUPRED2A disorder scores per residue for SLiMs (**D**), individual short IUPRED2A disorder scores per residue for SLiMs (**E**), and individual coil confidence scores per residue for SLiMs (**F**).

There were 32 SLiMs of the LIG_Rb_LxCxE_1 motif, 14 from viruses and 18 from eukaryotes. An analysis of the distribution of long and short IUPRED2A MIDS and MCCS values and long and short disorder and coil confidence per amino acid residue was performed. The LIG_Rb_LxCxE_1 motifs from viruses and eukaryotes show no significant difference in long and short MIDS, and short disorder score per residue values (Figure 10), but long disorder score per residue, MCCS values, and coil confidence per residue are all higher for eukaryotic LIG_Rb_LxCxE_1 motifs (*p*-value = 1.88 × 10^−3^, 3.30 × 10^−3^, and 0, respectively) (Figure 10). The majority of the coil confidence score per residue was above 0.8 in eukaryotes. In contrast, viruses showed a wide distribution of coil confidence per residue. These results suggest differences in the binding mechanism between LIG_Rb_LxCxE_1 instances from some viral proteins vs. eukaryotic proteins and that the eukaryotic LIG_Rb_LxCxE_1 motif in eukaryotes are found in disordered domains that are composed of a long sequence of amino acids, while viral proteins are less disordered and found in short disordered sequence regions.

The variation in coil confidence observed for viruses indicates that some viral LIG_Rb_LxCxE_1 instances have more secondary structure content than others and could demonstrate different affinity for Rb. Eukaryotes rely on transient interactions with the retinoblastoma proteins and a dynamic regulation of the cell cycle process where high affinity would be detrimental. Some viruses may be similar to eukaryotes, while others may display more of the secondary structure needed for the interaction to occur, resulting in higher affinity. Unlike eukaryotes, viruses would benefit from blocking the retinoblastoma protein from inhibiting transcription factor E2F to ensure the progression of the cell cycle [56,57]. 

**Figure 11:**
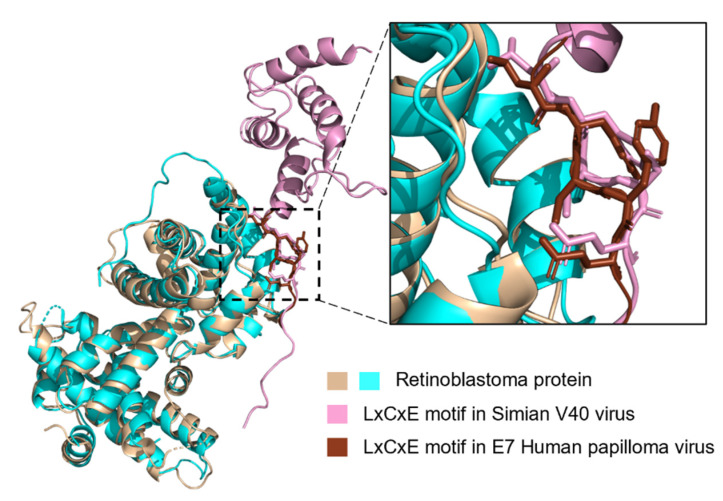
LIG_Rb_LxCxE_1 motif segment from Simian V40 (large T antigen protein) and human papillomaviruses (E7) proteins in a bound state with retinoblastoma protein. The complete structures from PDB ID: 1GH6 and PDB ID: 1GUX are aligned, and a closer view of the LxCxE binding site is shown. Retinoblastoma protein (beige and cyan) is rendered as a cartoon. Large T antigen protein is shown as a cartoon (dark pink). The E7 of the human papillomavirus motif segment is shown as ribbon (brown). The LxCxE motif in both proteins is shown as sticks. The structural alignment of the entire two structures was performed in PyMOL (PyMOL Molecular Graphics System, Version 4.6).

For the LxCxE motif, two instances from DNA viruses in the ELM database were annotated with a PDB structure; the large T antigen protein for Simian V40 virus (PDB ID: 1GH6 [58]) and E7 protein from human papillomavirus type 16 (PDB ID: 1GUX [59]). Neither structure represents the full-length proteins but instead truncated peptides of the LxCxE motif bound to human Rb. The secondary structure of the two motifs from the PDB structures based on DSSP reveals that the large T antigen protein is bound in a coil conformation, and the E7 protein is bound in a beta-strand conformation (Figure 11). Both motifs have high MCCS values of 0.94 and 0.95, respectively, and similar MIDS values (0.50 and 0.51, respectively) for long IUPRED2A prediction and similarly, MIDS values (0.64 and 0.65, respectively) for short IUPRED2A prediction. Previous research has shown that the E7 motif showed a lower Kd value than the Simian V40 T antigen protein and higher binding affinity towards the Rb protein than native eukaryotic proteins (reviewed in [51]). The low variability in high MCCS values for the eukaryotic motifs suggests that its flexibility is vital for its function. Furthermore, the differences in coil confidence and binding affinity of the LxCxE motifs in eukaryotic and viral proteins indicate a selection for high coil confidence and against high-affinity binding for Rb protein to maintain the transient regulatory binding inside the cells for eukaryotes. For viral proteins, these motifs may at first occur by chance in a near-neutral manner, but subsequent amino acid substitutions may improve Rb-binding and increase selective pressure to improve the strength of the interaction. 

## 3. Conclusions

Based on the currently available data from the ELM database, we have explored the potential differences in sequence-based structural features between true positive SLiMs in different taxonomic groups: eukaryotes, bacteria, and viruses. We find that viral SLiMs often are less disordered than SLiMs from eukaryotes and bacteria, which seems to stem from different ELM functionality type compositions across taxonomic groups rather than differences in disorder for the equivalent SLiM. For the same SLiMs, the disorder content is in good agreement across taxonomic groups, but exceptions exist. Proteins harboring SLiMs are overall less disordered in viruses than in eukaryotes and bacteria, but for all taxonomic groups, a peak in disorder is observed for the SLiM containing region based on short IUPRED2A prediction. For long IUPRED2A prediction, a small dip in disorder score is seen for SLiMs in eukaryotes as compared to the immediate flanking region but this dip is missing in viruses; however, overall, the SLiMs containing region is more disordered than the rest of the protein. We find that most SLiMs across all taxonomic groups in our study are devoid of secondary structure and instead have a loop or coil conformation. We analyzed coil confidence and found that proteins harboring SLiMs peak in coil confidence at the SLiM. While proteins from viruses again have lower coil confidence overall, high coil confidence and coil content describe most SLiMs. 

Disorder has been discussed as one of the most critical attributes in previous studies on SLiMs [5,25,60]. Our analysis of true positive SLiMs shows that classifying SLiMs as false positives based on their lack of disorder is not feasible. Based on the experimentally verified SLiMs in this study, classifying SLiMs based on coil confidence would yield better results. However, no comparison of true positives vs. actual false positive SLiMs could be completed due to lack of such data. While the current study did not investigate the evolutionary dynamics of pathogenic SLiMs, such studies including 3D structural features can bring further insights to molecular mimicry and host-pathogen interactions. 

We have illuminated characteristics of SLiMs that may play a role in how pathogens utilize molecular mimicry of SLiMs to alter the host cell machinery to their advantage. We find that SLiMs from pathogens occasionally present vastly different structural characteristics than the same SLiM in the host. It is plausible that molecular mimicry is mediated through a more limited set of conformations than in the host, and different mechanisms of binding cannot be ruled out. However, the dataset of equivalent SLiMs from eukaryotes and their pathogens is limited and biased towards certain ELM types. Another limitation stems from an uneven distribution of SLiMs from closely related homologs. If the ELM database contains the same motif from homologous conserved proteins, their characteristics can bias the results. When more data is available, this can be corrected for. An emphasis to experimentally verify more interactions involving SLiMs in pathogens is warranted to improve our understanding of molecular mimicry and host-pathogen interactions. In our analysis, we showed additional viruses that possess the same pattern for the MOD_N-GLC_1 motif from literature but not included in the ELM database. Unlike most MOD_N-GLC_1 entries from the ELM database, the additional instances were overall disordered. As more data becomes readily available, analyses and discoveries can be improved and enhanced methods for identifying host-pathogen PPI facilitated through molecular mimicry can be developed. Recent work by Wadie et al. applied structural and functional filters with information from viral SLiMs to enhance functional motif discovery in humans [61]. They used a low IUPRED2A disorder cutoff value of 0.2 to differentiate between functional and not functional SLiMs, but as we show here, caution must be taken when filtering viral SLiMs by IUPRED2A disorder even when using a very low cutoff value. A better understanding of the mechanistic differences displayed between the same SLiM in pathogens and their hosts holds promise for improving the utility of SLiMs as therapeutic drug targets. 

## 4. Methods

### 4.1. The ELM Dataset

The complete dataset of SLiM instances in the ELM database was downloaded on 10 October 2021. SLiMs annotated as True Positives were kept for further analysis. The taxonomic IDs for the organisms were extracted using NCBI taxonomy [62], and the True Positive (TP) instances were categorized according to their taxonomy: eukaryotes (taxonomic ID 2759), bacteria (taxonomic ID 2), and viruses (taxonomic ID 10239) and ELM type. SLiMs from eukaryotes and viruses were further divided into taxonomic subcategories. All complete protein sequences that harbor a SLiM were downloaded from the ELM database and used to extract the amino acid sequence for each instance based on the ELM regular expression patterns. The complete sequences were also used to generate sequence-based structural predictions for eukaryotes and bacteria. For viruses, since the downloaded data from the ELM database included polyproteins and not the individual proteins that contain the motif, a custom script was used to extract all the viral protein sequences including the ones in a polyprotein based on Uniprot database chain annotation. Some viral proteins did not have a chain annotation; these were manually examined and added to the viral dataset. For motifs that were found in-between two proteins based on UniProt chain annotation, the complete length of the two proteins were used. Three polyproteins that did not meet these criteria were excluded from the dataset.

### 4.2. Sequence-Based Structural Predictions

#### 4.2.1. Intrinsic Disorder Prediction

Intrinsic disorder propensity for the full-length SLiM containing proteins downloaded from the ELM database was predicted using the IUPRED2A webserver using both the default settings (IUPRED2A long disorder) and the IUPRED2A short disorder [63]. The long disorder option searches for long segments of disordered regions in proteins, while short disorder option searches for short segments in proteins that may have disorder property located in interdomain linkers or within domains. For each SLiM instance, both long and short disorder scores for its amino acids were extracted and used to calculate the percent of disorder per instance and the Mean IUPRED2A Disorder Score (MIDS). The percent disorder per instance was calculated based on how many residues were above a given cutoff divided by the total number of residues in the instance multiplied by 100. Two cutoffs, 0.4 and 0.5, respectively, were used. MIDS was calculated as the average disorder score for all residues per instance. 

#### 4.2.2. Relative Solvent Accessibility and Secondary Structure Predictions

A local installation of NetSurfP 2.0 [64] was used to predict relative solvent accessibility and secondary structure for all full-length SLiM containing proteins downloaded from the ELM database. Predictions were run using the HHblits method from the HHSuite [65] and uniclust30_2017_04 database [66]. Relative solvent accessibility was determined using a cutoff of 0.25. The coil or secondary structure assignment was considered based on the three-state prediction. For each instance, the percent of solvent-accessible and coil residues per instance were calculated as for the percent disorder described in Section 4.2.1. Coil confidence was extracted from the results and used to calculate the Mean Coil Confidence Score (MCCS) as for the average MIDS for all residues per instance. 

### 4.3. Phylogenetic Tree Analysis

To build the West Nile Virus (WNV) envelope protein phylogenetic tree, a protein BLAST [67] was done to determine homologous proteins using the NCBI accession of the WNV envelope protein (YP_001527877) that contain the MOD_N-GLC-1 motif. Extracted homologs of the protein were aligned with MAFFT using the L-INS-i setting [68] in Jalview [69]. IQ-Tree [70] using the default settings of automatic selection of the substitution model, branch support analysis using the ultrafast bootstrap method with default settings, and SH-alrt branch test with 1000 replicates were used to generate the tree. The tree was rooted on the outgroup virus (Yellow fever virus). The phylogenetic tree and the multiple sequence alignment were used to inspect the variability in sequence conservation and map the disorder and secondary structure properties onto the alignment, to allow exploring the differences in these properties between different clades visually. 

### 4.4. Statistical Analysis

Non-parametric statistical testing with Mann–Whitney was performed using a simplified Bonferroni multiple hypothesis testing correction (adjusted *p*-value = *p*-value multiplied by the number of tests, compared to alpha-value = 0.05) to infer statistically significant differences between groups. Spearman correlation analysis was performed to test the correlation between groups. Both tests were performed using the SciPy module [71]. 

## Data Availability

Data, scripts, and Jupyter notebooks used for the analysis can be accessed at https://github.com/Heidy-Elkhaligy/Comparative-Analysis-of-Structural-Features-in-SLiMs-from-Eukaryotes-Bacteria-and-Viruses.git.

## Supplementary Materials

The following supporting information can be downloaded at: https://www.mdpi.com/article/10.3390/pathogens11050583/s1, Figure S1: Scatter plot for long disorder vs short disorder MIDS per instance; Figure S2: Scatter plot for eukaryotes long disorder vs short disorder MIDS per instance; Figure S3: Scatter plot for bacteria long disorder vs short disorder MIDS per instance; Figure S4: Scatter plot for viruses long disorder vs short disorder MIDS per instance; Figure S5: Phylogenetic tree of West Nile Virus envelope protein rooted by the outgroup Yellow Fever virus (YFV); Table S1: ELM dataset, prediction, and analysis; Table S2: SLiMs count and percentage per ELM type in eukaryotes, bacteria and viruses; Table S3: Disorder and coil confidence profile data for 100 residues around the SLiM; Table S4: Shared motifs prediction data between taxonomic groups.

## Abbreviations

PPI	Protein-protein interactions
ELM	Eukaryotic Linear Motifs
SLiMs	Short Linear Motifs
IDR	Intrinsically Disordered protein Region
IDP	Intrinsically Disordered Protein
MIDS	Mean IUPRED2A Disorder Score
MCCS	Mean Coil Confidence Score
mMIDS	mean MIDS
mMCCS	mean MCCS
LIG	Ligand binding motifs
MOD	Post-translational modification motifs
TRG	Targeting motifs
DOC	Docking motifs
CLV	Cleavage sites motifs
DEG	Degradation motifs
WNV	West Nile Virus
Rb	Retinoblastoma protein
DSSP	Dictionary of Secondary Structure of Proteins
PDB	Protein Data Bank
