# Supplementary material for: Comparative Analysis of Structural Features in SLiMs from Eukaryotes, Bacteria, and Viruses with Importance for Host-Pathogen Interactions
Source: Pathogens. 2022 May 15;11(5):583. doi: 10.3390/pathogens11050583 (PMC9147284; doi:10.3390/pathogens11050583)
Supplement: Supplementary file 1 [file pathogens-11-00583-s001.zip › pathogens-1665034-supplementary/supplementary_figures_final.pdf]

Supplementary figures

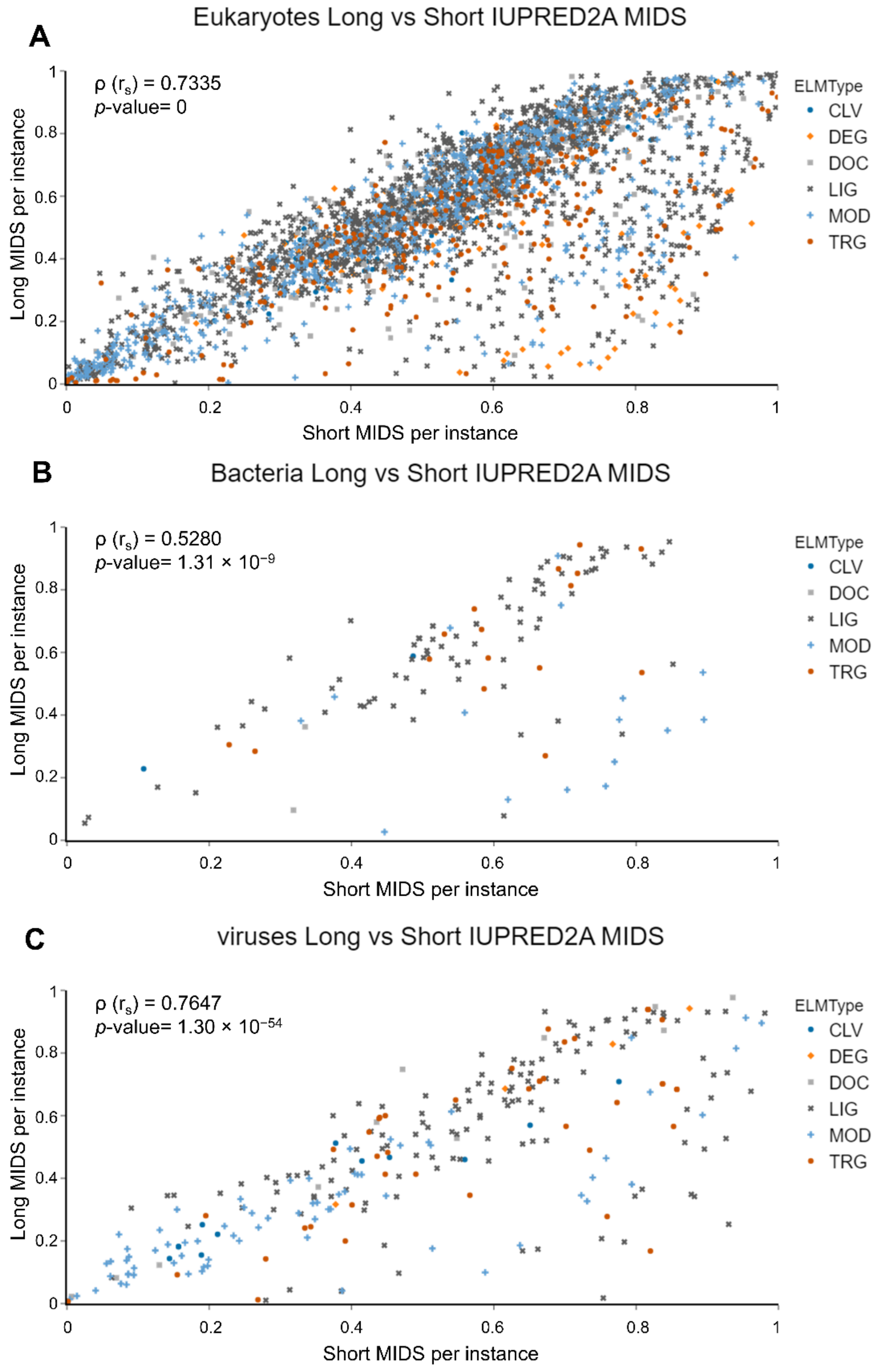

Figure S1: Scatter plot for long disorder vs short disorder MIDS per instance. For eukaryotes (A), bacteria (B), and viruses (C). The Spearman correlation and p-value is shown in each plot. An interactive version of this plot can be generated with Plotly using the Jupyter notebooks included with this publication.

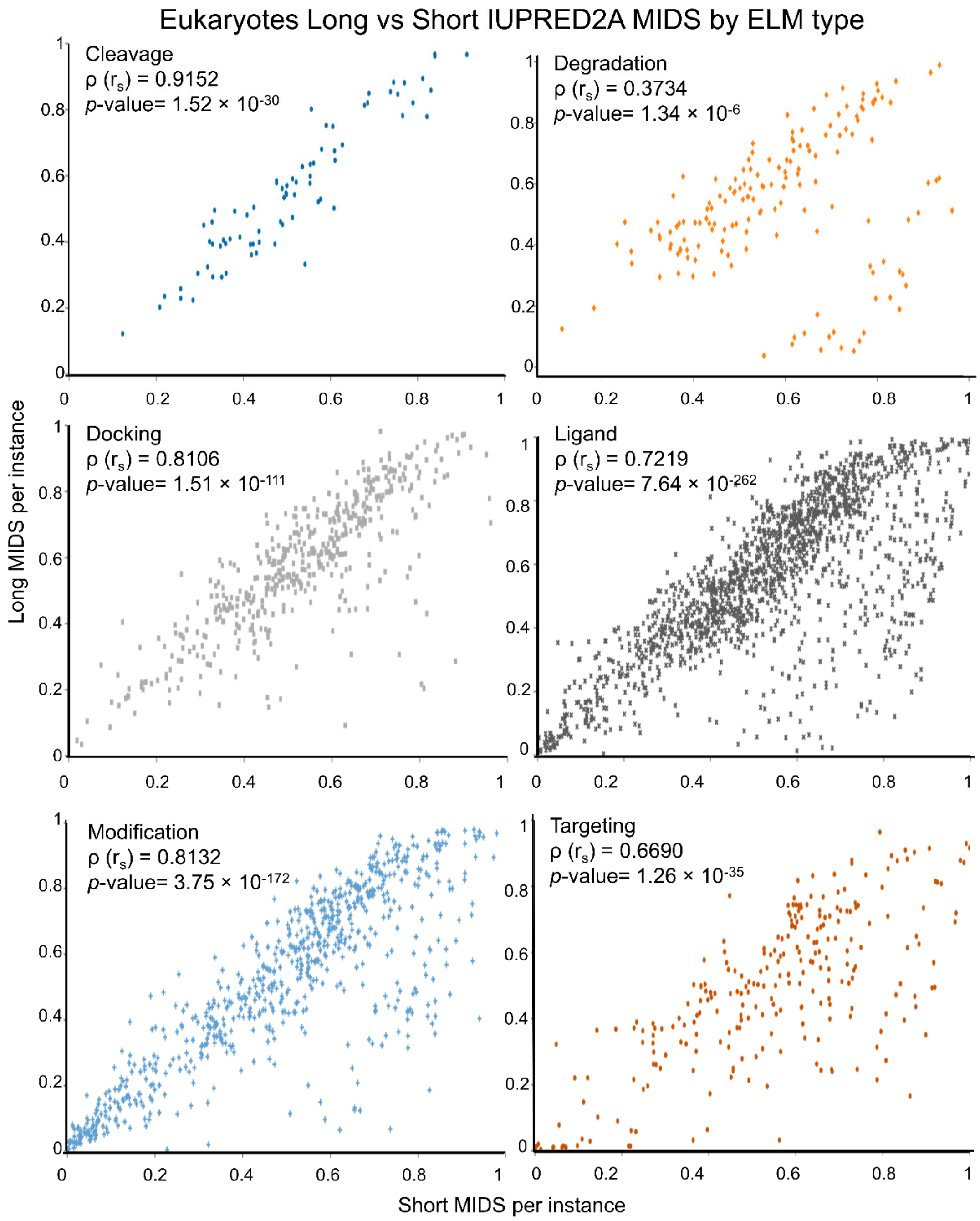

Figure S2: Scatter plot for eukaryotes long disorder vs short disorder MIDS per instance. The Spearman correlation and p-value for the total instances and per ELM type is shown in each plot. An interactive version of this plot can be generated with Plotly using the Jupyter notebooks included with this publication.

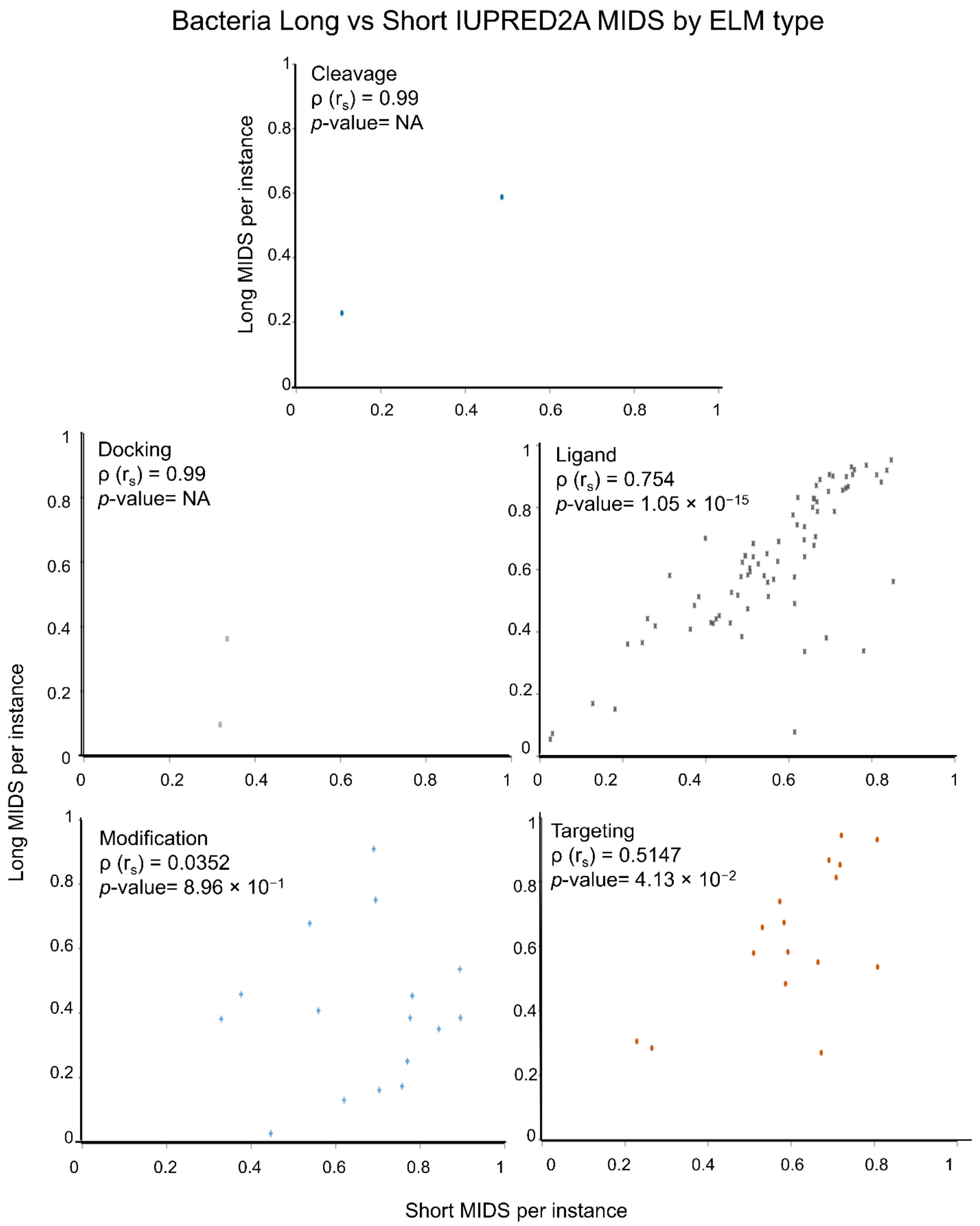

Figure S3: Scatter plot for bacteria long disorder vs short disorder MIDS per instance. The Spearman correlation and p-value for the total instances and per ELM type is shown in each plot. An interactive version of this plot can be generated with Plotly using the Jupyter notebooks included with this publication.

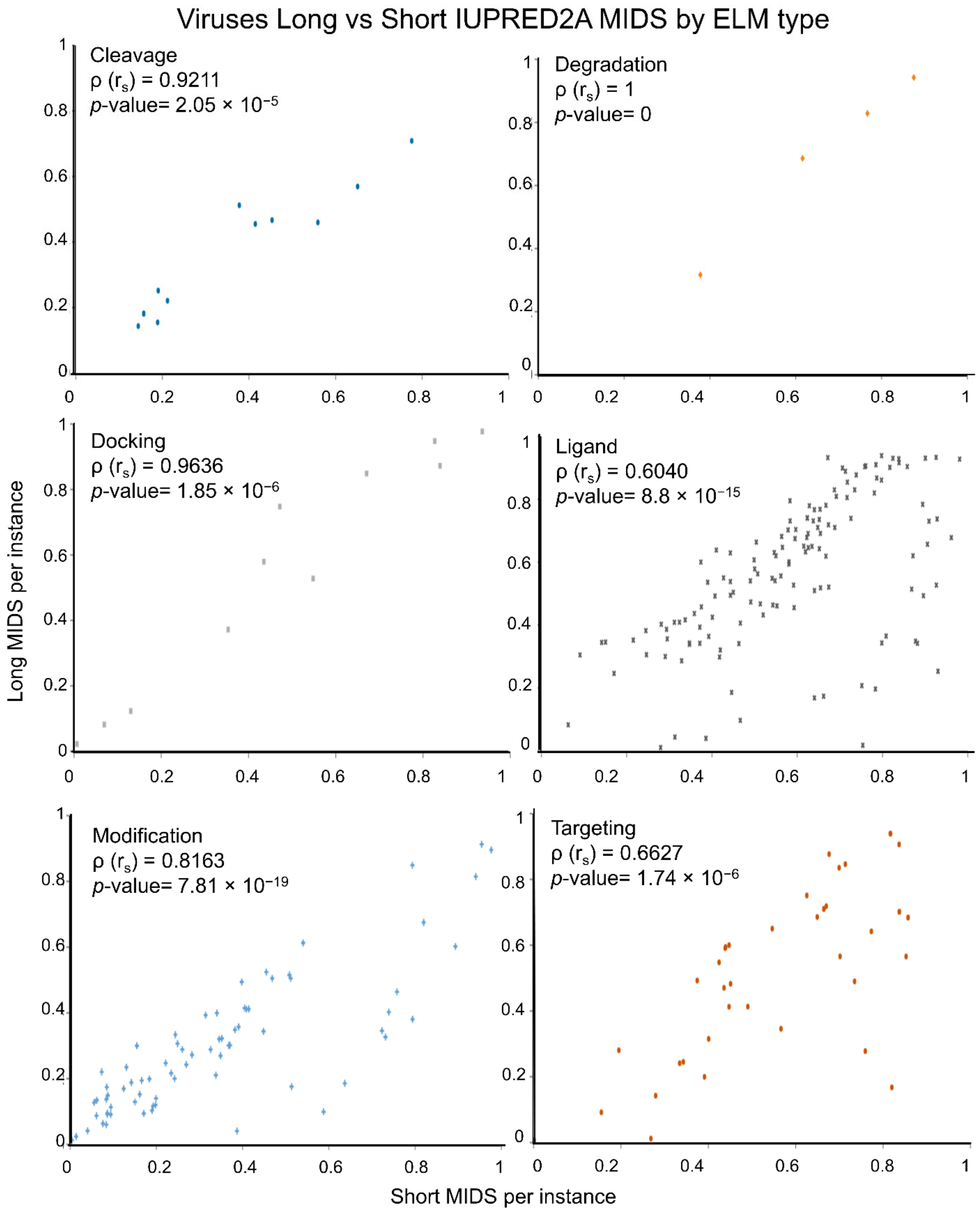

Figure S4: Scatter plot for viruses long disorder vs short disorder MIDS per instance. The Spearman correlation and p-value for the total instances and per ELM type is shown in each plot. An interactive version of this plot can be generated with Plotly using the Jupyter notebooks included with this publication.

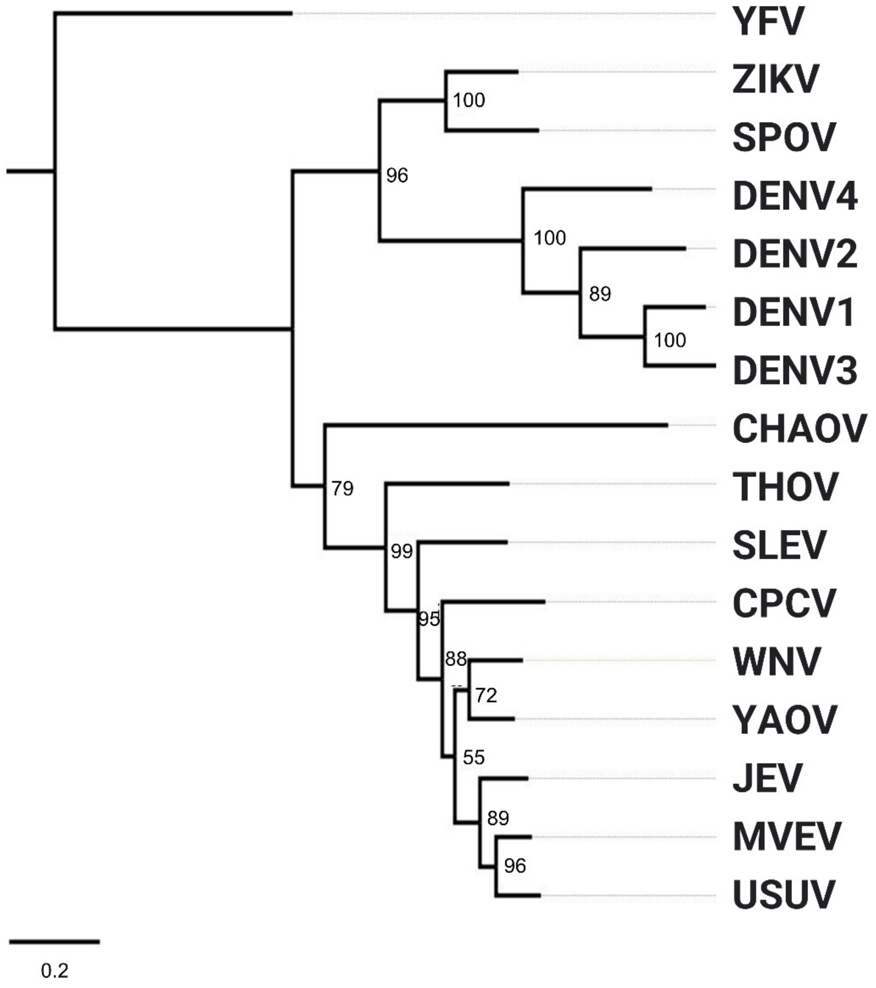

Figure S5: Phylogenetic tree of West Nile Virus envelope protein rooted by the outgroup Yellow Fever virus (YFV). Bootstrap support based on Ultrafast boostraps from IQ-Tree are shown. Envelope proteins included correspond to WNV (West Nile Virus, accession: YP_001527877.1, range: 291-791), YAOV (Yaounde virus, accession: YP_009350103.1, range: 291-791), JEV (Japanese encephalitis virus, accession: NP_775666.1, range: 1-500), MVEV (Murray Valley encephalitis virus, accession: NP_722531.1, range: 1-501), USUV (Usutu virus, accession: YP_164819.1, range: 1-500), CPCV (Cacipacore virus, accession: YP_009126874.1, range: 289-789), SLEV (Saint Louis encephalitis virus, accession: YP_009329949.1, range: 1-501), THOV (T'Ho virus, accession: YP_009351820.1, range: 287-787), ZIKV (Zika virus, accession: YP_009430300.1, range: 1-504), SPOV (Spondweni virus, accession: YP_009227187.1, range: 3-503), CHAOV (Chaoyang virus, accession: YP_009259657.1, range: 1-492), DENV1 (Dengue virus 1, accession: NP_722460.2, range: 1-495), DENV4 (Dengue virus 4, accession: NP_740317.1, range: 1-495), DENV2 (Dengue virus 2, accession: NP_739583.2, range: 1-495), DENV3 (Dengue virus 3, accession: YP_001531168.2, range: 1-493), and YFV (Yellow fever virus, accession: NP_740305.1, range: 2-493).
